# G-Protein-Coupled Estrogen Receptor-1 Positively Regulates the Growth Plate Chondrocyte Proliferation in Female Pubertal Mice

**DOI:** 10.3389/fcell.2021.710664

**Published:** 2021-08-20

**Authors:** Ya-Shuan Chou, Shu-Chun Chuang, Chung-Hwan Chen, Mei-Ling Ho, Je-Ken Chang

**Affiliations:** ^1^Orthopaedic Research Center, Kaohsiung Medical University, Kaohsiung, Taiwan; ^2^Regenerative Medicine and Cell Therapy Research Center, Kaohsiung Medical University, Kaohsiung, Taiwan; ^3^Department of Orthopaedics, Kaohsiung Medical University Hospital, Kaohsiung Medical University, Kaohsiung, Taiwan; ^4^Department of Orthopaedics, College of Medicine, Kaohsiung Medical University, Kaohsiung, Taiwan; ^5^Department of Orthopaedics, Kaohsiung Municipal Ta-Tung Hospital, Kaohsiung, Taiwan; ^6^Institute of Medical Science and Technology, National Sun Yat-sen University, Kaohsiung, Taiwan; ^7^Department of Healthcare Administration and Medical Informatics, Kaohsiung Medical University, Kaohsiung, Taiwan; ^8^Department of Physiology, College of Medicine, Kaohsiung Medical University, Kaohsiung, Taiwan; ^9^Department of Marine Biotechnology and Resources, National Sun Yat-sen University, Kaohsiung, Taiwan; ^10^Department of Medical Research, Kaohsiung Medical University Hospital, Kaohsiung Medical University, Kaohsiung, Taiwan

**Keywords:** G-protein-coupled estrogen receptor-1, chondrocyte-specific knockout mice, estrogen receptor, bone growth, long bone elongation

## Abstract

Estrogen enhances long bone longitudinal growth during early puberty. Growth plate chondrocytes are the main cells that contribute to long bone elongation. The role of G-protein-coupled estrogen receptor-1 (GPER-1) in regulating growth plate chondrocyte function remains unclear. In the present study, we generated chondrocyte-specific GPER-1 knockout (CKO) mice to investigate the effect of GPER-1 in growth plate chondrocytes. In control mice, GPER-1 was highly expressed in the growth plates of 4- and 8-week-old mice, with a gradual decline through 12 to 16 weeks. In CKO mice, the GPER-1 expression in growth plate chondrocytes was significantly lower than that in the control mice (80% decrease). The CKO mice also showed a decrease in body length (crown–rump length), body weight, and the length of tibias and femurs at 8 weeks. More importantly, the cell number and thickness of the proliferative zone of the growth plate, as well as the thickness of primary spongiosa and length of metaphysis plus diaphysis in tibias of CKO mice, were significantly decreased compared with those of the control mice. Furthermore, there was also a considerable reduction in the number of proliferating cell nuclear antigens and Ki67-stained proliferating chondrocytes in the tibia growth plate in the CKO mice. The chondrocyte proliferation mediated by GPER-1 was further demonstrated *via* treatment with a GPER-1 antagonist in cultured epiphyseal cartilage. This study demonstrates that GPER-1 positively regulates chondrocyte proliferation at the growth plate during early puberty and contributes to the longitudinal growth of long bones.

## Introduction

Long bone longitudinal growth is mainly driven by chondrocyte proliferation at the growth plate during puberty. Estrogen is well-known to regulate longitudinal growth during puberty (Chagin and Savendahl, 2007a). More importantly, low estrogen levels stimulate bone growth in early puberty, whereas high estrogen levels induce growth plate closure at the end of puberty (Almeida et al., 2017). The molecular mechanisms involved in the change of estrogen levels and the differences of estrogen receptors (ERs) remain unclear. A membranous ER, G-protein-coupled estrogen receptor-1 (GPER-1), also called GPR30, was recently shown to mediate the non-genomic effects of estrogen (Revankar et al., 2005). GPER-1 has been indicated to be widely expressed in mouse and human tissues, such as the heart (Martensson et al., 2009; Patel et al., 2010), brain (Hazell et al., 2009), pancreas (Liu et al., 2009; Martensson et al., 2009; Kumar et al., 2011), uterus (Gao et al., 2011), bone (Chagin and Savendahl, 2007b; Heino et al., 2008), and cartilage (Chagin and Savendahl, 2007b; Ribeiro et al., 2020). More importantly, the expression level of GPER-1 in the human growth plate was found to decrease during pubertal progression, suggesting that GPER-1 might be involved in the modulation of pubertal bone growth (Chagin and Savendahl, 2007b). Although GPER-1 expression in the bone and cartilage has been investigated previously, the function of GPER-1 in bone growth remains unclear. Accordingly, in this study, we hypothesized that GPER-1 might regulate early growth plate development and affect long bone longitudinal growth.

In global *GPER-1* knockout mice, GPER-1 deficiency causes certain metabolic alterations as well as a reduction in body weight and bone growth, suggesting that GPER-1 might play a role in skeletal development (Martensson et al., 2009). In contrast, another study showed that the increase in body weight in female global *GPER-1* knockout mice was due to abnormal obesity (Haas et al., 2009). However, these studies using global *GPER-1* knockout mice did not specifically investigate the role of GPER-1 in growth plate chondrocytes and long bone longitudinal growth. Recently, the Cre/loxP system has been used to generate a tissue-specific *GPER-1* knockout mouse model, serving as an alternative experimental strategy and providing a more reliable phenotype. In this study, we developed a chondrocyte-specific *GPER-1* deficient (*Col2a1-Cre*; *GPER-1^*f/f*^*, CKO) mouse model to investigate the role of GPER-1 in the growth plate chondrocytes of growing bones. The role of GPER-1 in the regulation of longitudinal bone growth during puberty, including bone length, growth plate thickness, and growth plate chondrocyte proliferation in long bones, was investigated using the animal model.

## Materials and Methods

### Experimental Animals

All animal studies were approved by the Kaohsiung Medical University Animal Care and Use Committee (104166 and 107157). Four animals were housed per cage, maintained on a 12/12-h light/dark cycle at 23 ± 2°C, with food and water freely available (Altromin, DEU).

In female mice, the onset of puberty can occur as early as P26 (3.5 weeks old) (Bell, 2018), and the age of mature adult mice can range from 3 to 6 months (Flurkey et al., 2007). Therefore, according to previous reports, 2-, 4-, 8-, 12-, and 16-week-old mice represent the stages of early life, early-puberty, puberty, end-puberty, and post-puberty, respectively (van der Eerden et al., 2000; Li et al., 2012). The body weight of the mice was analyzed every week, and axial growth was measured through the crown–rump length before death. Mice were randomly killed at 2, 4, 8, 12, and 16 weeks old (*n* = 6–8 mice per group), at which point the tissues were isolated. Bone tissues were collected and fixed with a 10% formalin solution, decalcified in a 10% ethylenediaminetetraacetic acid solution, embedded in paraffin, and sectioned at a thickness of 5 μm.

### Generation of *Col2a1-Cre*; *GPER-1^*f/f*^* Mice

The *GPER-1^tm 1*c*^* conventional GPER-1 mice were purchased from the Knockout Mouse Project (University of California-Davis, Davis, CA, United States). The *GPER-1^tm 1*c*^* mice were generated by crossing the *GPER-1^tm 1*a*^* mice with Flp mice, which ubiquitously express Flp recombinase. The flanking loxP sites within exon 3 were generated, expanded, and injected into the C57BL/6 blastocysts as part of the Knockout Mouse Project (Skarnes et al., 2011). The offspring were crossed with flippase-transgenic mice to remove the NeoR flanked by flippase recombinase target sequences. The hybrid mice were backcrossed with the C57BL/6 strain for 12 generations. The *GPER-1^tm 1*c*^* mice were generated and further maintained with a C57BL/6J background.

To generate the chondrocyte-specific (*Col2a1*-Cre) homozygous floxed GPER-1 transgenic (*Col2a1*-Cre; *GPER-1^*f/f*^*) mice, the *GPER-1^tm 1*c*^* mice were crossed with *Col2a1*-Cre mice, which were purchased from the Jackson Laboratory (JAX stock #003554; Bar Harbor, ME, United States) (Ovchinnikov et al., 2000) to obtain the offspring *Col2a1*-Cre; *GPER-1^+/^*^*f*^ mice. The resulting offspring were then intercrossed to breed chondrocyte-specific *GPER-1* knockout mice, *Col2a1-*Cre; *GPER-1^*f/f*^* mice (*n* = 28). To confirm the genotypes of these offspring, genomic DNA was obtained from the tails of the mice. Genotyping of the GPER-1 floxed allele was performed by polymerase chain reaction (PCR) using the forward and reverse primers 5′-GAA CCC ACA GCT CTC TTG TGT GC-3′ and 5′-GGA AAA CTA CTG TTT GTC GAG ACA GG-3′, which amplified a 507-bp fragment, whereas the GPER-1 wild-type allele produced a 322-bp fragment. Moreover, the *Col2a1*-Cre transgene was detected by PCR using the forward and reverse primers 5′-CTA AAC ATG CTT CAT CGT CGG TC-3′ and 5′-TCG GAT CAT CAG CTA CAC CAG AG-3′, which produced a 420-bp fragment. In this study, the *GPER-1^*f/f*^* mice without *Col2a1-*Cre were used as the control group (*n* = 34). All animals were generated in the National Laboratory Animal Center (Tainan, Taiwan).

### Measurement of Serum Estrogen Levels

Blood samples were collected from the proestrus vena cava of 8-week-old mice (*n* = 4). To quantitatively detect estrogen in CKO or control mouse serum, a mouse estrogen enzyme-linked immunosorbent assay (ELISA) kit (EM1501, FineTest, Hubei, China) was used. First, 50 μl of standard or sample was added to each well that had been pre-coated with estrogen. Second, 50 μl biotin-detection antibody was added to each well of 96-well plate and was incubated for 45 min at 37°C. The antibody was removed and washed using wash buffer, and then, 100 μl SABC working solution was added to each well for 30 min at 37°C. After the working solution was removed, 90 μl of 3,3′,5,5′-tetramethylbenzidine substrate was added, and the solution was incubated for 15 min at 37°C. Finally, a stop solution was added, and the absorbance of the samples was measured using an ELISA reader at 450 nm. For the estrogen ELISA kit, a standard curve was generated to calculate the value of the tested sample in each assay. The testing sensitivity was 15.625 pg/ml, the concentration range was 15.625 to 1,000 pg/ml, and the mean ± SD deviation of the *R*^2^ value was 0.99 ± 0.0047 for all assays. The intra-assay and inter-assay coefficients of variation (CV) were 6.79% (*n* = 9) and 9.08% (*n* = 3), respectively. The data corresponded with the criteria (intra-assay: CV < 8%, and inter-assay: CV < 10% from the protocol).

### Micro-Computed Tomography Imaging System for Bone Structure Analysis

Three-dimensional (3-D) reconstruction of the specimens was performed using high-resolution micro-computed tomography (μ-CT) analysis (Skyscan 1076; Skyscan NV, Kontich, Belgium) to characterize bone formation at the ultrastructural level in more detail. Mice (*n* = 4–8) were anesthetized and scanned at an isotropic voxel resolution of 18 μm with a 0.5 mm aluminum filter, a 50-kV X-ray tube voltage, a 200 μA tube electric current, and a 520-ms scanning exposure time. The 3-D images were reconstructed for analysis using a scale of 0–0.065 (NRecon version 1.6.1.7; Skyscan NV, Kontich, Belgium). The 3-D morphometric parameters were computed using the direct 3-D approach, including the lengths of the tibia, femur, epiphysis, and metaphysis plus diaphysis, and thicknesses of the cortical bone, growth plate, and primary spongiosa (in millimeter). The region of interest from the 3-D reconstruction images was obtained and analyzed using CTAn software (CT-Analyser version 1.20.3.0; Skyscan NV, Kontich, Belgium). To measure the tibia length, we determined the distance between the proximal end of the tibia and the most distal end of the medial malleolus. To measure the femur length, we calculated the distance between the proximal end of the femoral head and the most distal end of the condyle. The cortex thickness of the diaphysis was measured using a cross-sectional view of the 3-D reconstruction image for a 2-mm segment at the mid-diaphysis. The thicknesses of the growth plate and the primary spongiosa were reported as the mean values measured from 30 sites in the mid-coronal section of each proximal tibia.

### Safranin O/Fast Green Staining for the Observation of Growth Plate Histology

Sulfated glycosaminoglycan was stained with Safranin O/Fast Green (1% Safranin-O counter-stained with 0.75% hematoxylin and 1% Fast Green; Sigma-Aldrich, St Louis, MO, United States). The histological measurements (tissue section, *n* = 8) were performed at the central three-fourths of the growth plate sections using the Image J software (National Institutes of Health, Bethesda, MD, United States). The average cell numbers were calculated from three independent visual fields per growth plate. The average heights of the growth plate, resting zone, proliferative zone, and hypertrophic zone were the mean values measured from 15 sites in each growth plate.

### Immunohistochemistry for Detecting Protein Expression in Growth Plate Cartilage

The fixed sections of the tibia, rib, and uterus were pretreated with the antigen retrieval solution suppressing all endogenous peroxidase activity and incubated with primary antibodies at 4°C overnight (*n* = 5). The following antibodies were used in this study: GPER-1 (Santa Cruz Biotechnology, Dallas, TX, United States), estrogen receptor-alpha (ERα) (Santa Cruz Biotechnology), type II collagen (Abcam, Cambridge, MA, United States), type X collagen (St John’s laboratory, London, United Kingdom), proliferating cell nuclear antigen (PCNA) (Abcam), and Ki67 (Millipore, Burlington, MA, United States). Peroxidase-conjugated anti-mouse or anti-rabbit antibodies (Santa Cruz Biotechnology) were used as the secondary antibody and were visualized by 3,3′-diaminobenzidine staining. The tissues were stained with hematoxylin (Sigma-Aldrich, St Louis, MO, United States) to visualize the nuclei, and the images were observed and photographed using a microscope (Nikon, Japan). Immunohistochemistry (IHC) measurements were performed at the central three-fourths of the growth plate sections using the Image J software (National Institutes of Health, Bethesda, MD, United States). The number of 3,3′-diaminobenzidine-positive cells was calculated from three independent visual fields per growth plate. The thicknesses of the type X collagen-stained hypertrophic zone were the mean values measured from 15 sites on each growth plate.

### Bromodeoxyuridine Assay to Assess Cell Proliferation in Cultured Epiphyseal Cartilage

G1 is a specific agonist, whereas G15 is a specific antagonist of GPER-1, both of which have no effect on classic ERs (Bologa et al., 2006; Dennis et al., 2009). G-1 (881639-98-1; Cayman Chemical, MI, United States) and G-15 (1161002-05-6; Cayman Chemical) were dissolved in dimethyl sulfoxide as a stock solution. Four-day-old neonatal rats were killed, and their tibias were harvested (*n* = 5). The samples were washed three times with antibiotics to avoid contamination, and part of the epiphyseal cartilage was harvested. The samples were cultured in BGJb medium containing 10% charcoal-striped serum (100-ml fetal bovine serum treated with 0.5-g charcoal and 0.052-g dextran T-70 for 2 h at 37°C and then centrifuged at 12,000 rpm for 10 min), and 0.5% antibiotics.

The cultured cartilages were divided randomly into the control, G-1, and G15 treatment groups (*n* ≥ 5). Approximately 6 days after treatment, 10 μM bromodeoxyuridine (BrdU) labeling solution (Abcam) was added to each group, and the cultured cartilages were further incubated for 24 h at 37°C. After BrdU treatment for 24 h, the samples were harvested and fixed with 10% neutral buffered formalin. The cultured cartilages were decalcified in 10% formic acid and embedded in paraffin, and 5 μm-thick sections were prepared and processed according to the instructions provided in the BrdU IHC Kit (ab125306; Abcam, Cambridge, MA, United States). The number of BrdU-positive cells was counted from three independent visual fields per cultured epiphyseal cartilage using Image J software (National Institutes of Health, Bethesda, MD, United States).

### Statistical Analyses

Each experimental group was repeated with at least five mice, and these data are expressed as the mean ± standard deviation. Statistical analyses were performed using the SPSS 20 (Chicago, IL, United States) software. Data were visualized using box plots with the median as represented by the SigmaPlot version 12 (San Jose, CA, United States) software. All data points are displayed. For comparisons between two groups, the *P*-values were calculated using either the paired or the unpaired two-tailed Student’s *t*-tests. The one-way analysis of variance evaluated statistical significance, and multiple comparisons were performed using Scheffé’s method for three-group statistical analyses. *P*-values <0.05 were considered statistically significant.

## Results

### Age-Related Changes of G-Protein-Coupled Estrogen Receptor-1 and Estrogen Receptor-Alpha Expressions in Tibia Growth Plates

**FIGURE 1:**
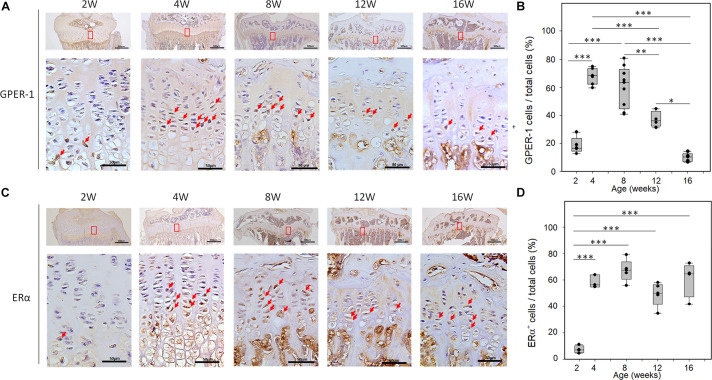
Age-related changes of GPER-1 and ERα levels in tibia growth plates as determined *via* IHC staining. **(A,C)** Growth plate cartilage of tibias from 2-, 4-, 8-, 12-, and 16-week-old mice were stained for GPER-1 and ERα. Representative micrographs of growth plates at low (scale bars, 500 μm) and high (scale bars, 50 μm) magnification. **(B,D)** Image-J analysis of percentage of GPER-1- and Erα-positive cells from total number of hematoxylin-stained cells (total cells). Each group, *N* = 5. ^∗^*P* < 0.05, ^∗∗^*P* < 0.01, ^∗∗∗^*P* < 0.001.

The distribution of the GPER-1 protein was detected on the sections of the tibial growth plates from the female mice. The immunoreactivity of GPER-1 in growth plate chondrocytes was detected in 2-, 4-, 8-, 12-, and 16-week-old mice (Figure 1A). In 2-week-old mice, the growth plate chondrocytes showed minimal GPER-1 expression. In early puberty, the GPER-1 expression was significantly increased in 4-week-old mice compared with that in 2-week-old mice. In the growth plate chondrocytes of 4- and 8-week-old mice, 68.69 ± 5.90% and 60.61 ± 14.45% of the cells were GPER-1 positive, respectively. During the end-puberty stage in 12-week-old mice, GPER-1 expression decreased to 37.2 ± 5.68%. After sexual maturation in 16-week-old mice, the values decreased to less than 10%. The quantitative analysis of GPER-1-positive cells showed an age-related variation in the tibial growth plate of mice (Figure 1B).

Developmental changes in ERα expression were also observed in the tibia of female mice. In 2-week-old mice, minimal ERα expression was observed in the growth plate chondrocytes. During puberty, abundant cellular staining of ERα was observed in the growth plate (Figure 1C). ERα immunoreactivity was detected in the resting, proliferative, and hypertrophic chondrocytes of 4- to 16-week-old mice. However, ERα expression did not show significant age-related variation in these mice (Figure 1D).

### Generation of the Chondrocyte-Specific *G-Protein-Coupled Estrogen Receptor-1* Knockout (CKO) Mice

**FIGURE 2:**
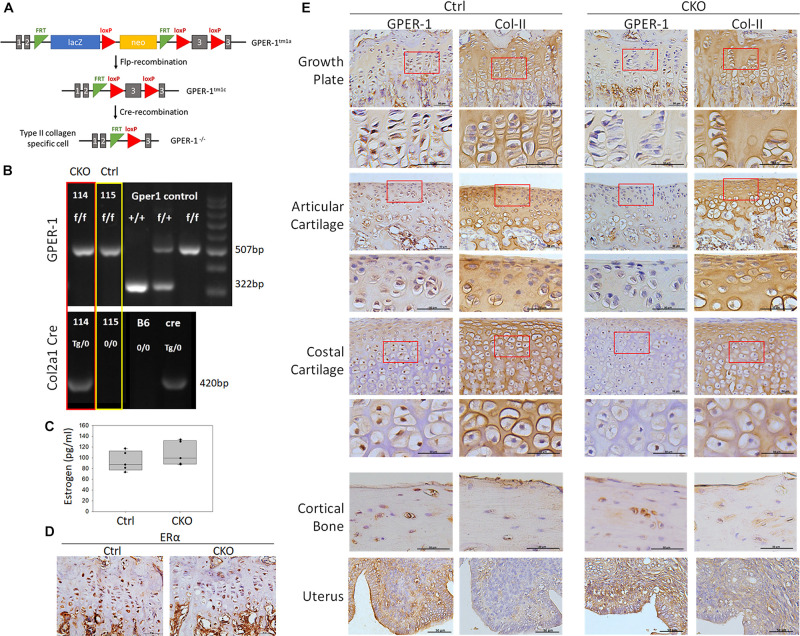
Generation of chondrocyte-specific GPER-1 knockout (CKO) mice using Cre/loxP system. **(A)** Schematic diagram of producing *Col2a1-Cre*; *GPER-1^*f/f*^* mice. **(B)** Genotyping of *Col2a1-Cre* and *GPER-1* in control (Ctrl) and CKO mice. Genotyping of GPER-1 floxed allele amplified a 507-bp fragment, whereas GPER-1 wild-type allele produced a 322-bp fragment. *Col2a1-Cre* transgene produced a 420-bp fragment. Each group, *N* = 28–34. **(C)** Serum levels of estrogen showed no significant difference with GPER-1 deficiency. **(D)** IHC staining of ERα in tibia growth plates showed no significant difference between two groups. Scale bars, 50 μm. **(E)** GPER-1 and type II collagen (Col-II) were stained by IHC staining and analyzed in tibia growth plate, articular cartilage, costal cartilage, cortical bone, and uterus. Representative micrographs of growth plates at low and high magnification. Scale bars, 50 μm. Each group, *N* = 3–5.

To elucidate GPER-1-mediated chondrocyte functions, we generated a CKO mouse model with a floxed exon 3 at the *GPER-1* locus, which is the only coding exon of the *GPER-1* gene (Figure 2A). In the CKO mice, Cre was expressed only in chondrocytes, which expressed type II collagen. Genotyping was performed by PCR using tail genomic DNA (Figure 2B). Both the control and CKO groups had *GPER-1* floxed alleles (507 bp). Only CKO mice expressed Cre recombinase under the type II collagen-specific promoter (420 bp). There was no change in the serum estrogen levels between the control and CKO mice (Figure 2C). Furthermore, ERα expression in the tibia growth plates showed no significant difference between the control and CKO mice as verified *via* IHC staining (Figure 2D).

Chondrocyte-specific GPER-1 knockout mice had a reduced GPER-1 protein expression in chondrocytes but not in the cortical bone tissue or the uterus (Figure 2E). IHC of the growth plate cartilage, articular cartilage, and costal cartilage demonstrated GPER-1 immunoreactivity in type II collagen-positive cells in control mice, which were almost completely abolished in CKO mice. In the growth plate cartilage, the number of GPER-1 positive cells decreased by approximately 82% in CKO mice.

### Phenotypical Changes in the Body Weight, Length, Bone Length, and Cortical Bone Thickness in Chondrocyte-Specific G-Protein-Coupled Estrogen Receptor-1 Knockout Mice

**FIGURE 3:**
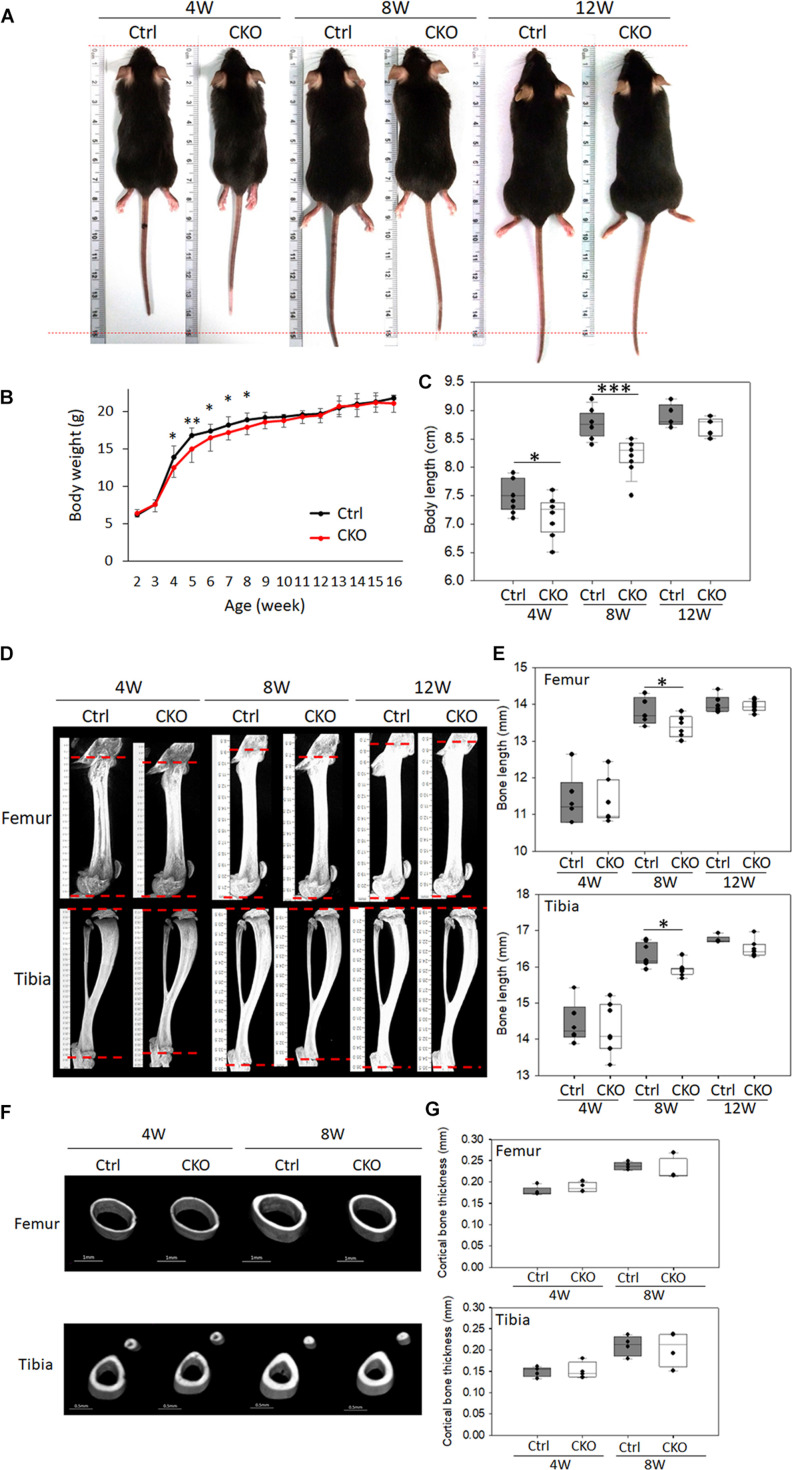
Comparison of phenotypes between chondrocyte-specific GPER-1 knockout mice (CKO) and control mice (Ctrl). **(A)** General appearance of 4-, 8-, and 12-week-old female mice. **(B)** Body weight of CKO and Ctrl mice. **(C)** Body length (crown–rump length) of CKO and Ctrl mice. **(D,E)** Femur and tibia μ-CT images and length quantitation in 4-, 8-, and 12-week-old CKO and Ctrl mice. **(F,G)** Cortical bone thicknesses were analyzed *via* μ-CT. Each group, *N* = 6–8. ^∗^*P* < 0.05, ^∗∗^*P* < 0.01, ^∗∗∗^*P* < 0.001.

The CKO mice exhibited a short body phenotype compared with the control mice (Figure 3A). The CKO mice had decreased body weight (Figure 3B) as well as shorter body length (crown–rump length) at 4 and 8 weeks than the control mice (Figure 3C). The femoral length of CKO mice decreased by 2.84%, and the tibial length decreased by 2.32% in 8-week-old mice compared with that in the control mice, as determined *via* μ-CT analysis (Figures 3D,E). The cortex thickness was analyzed in the middle of the diaphysis, and there was no difference between the CKO and control mice (Figures 3F,G).

These findings indicated that the reduced body weight might be associated with a reduction in bone growth because both the axial and appendicular skeletons were significantly shortened in the 8-week-old CKO mice. The chondrocyte-specific GPER-1 deficiency may regulate endochondral ossification rather than intramembranous ossification, as there were skeletal changes in bone length rather than cortical bone thickness.

### Changes of Growth Plate Development in the Chondrocyte-Specific G-Protein-Coupled Estrogen Receptor-1 Knockout Mice

**FIGURE 4:**
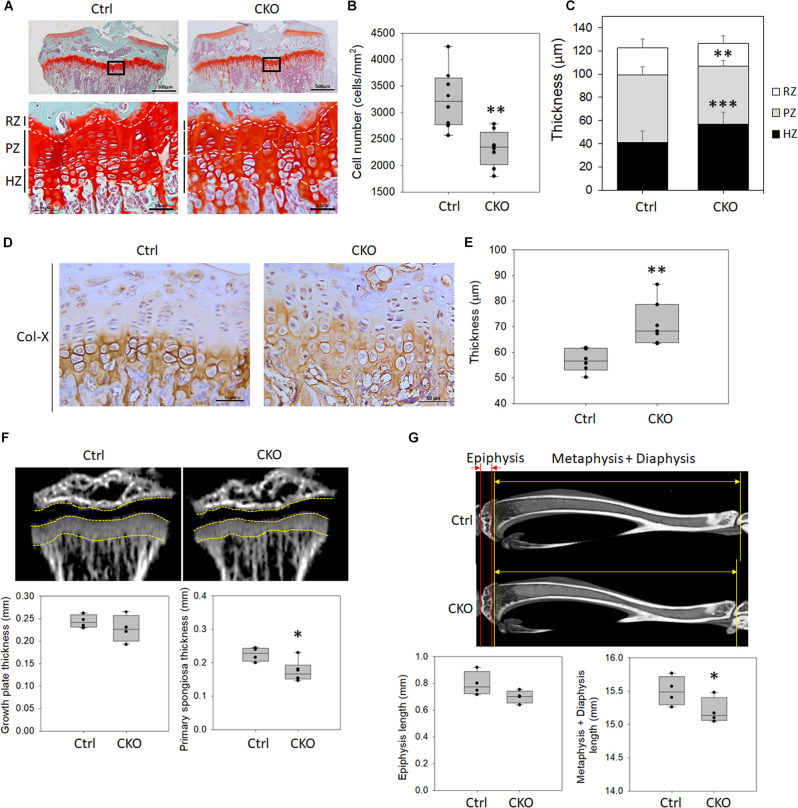
Changes of growth plate development in chondrocyte-specific GPER-1 knockout (CKO) mice. **(A)** Tibial growth plates of 8-week-old female mice were stained with Safranin O/Fast Green staining. Representative micrographs of growth plates at low (scale bars, 500 μm) and high (scale bars, 50 μm) magnification. **(B)** Cell numbers were measured in CKO mice. **(C)** Thicknesses of resting zone, proliferation zone, and hyperopic zone were analyzed. **(D)** Hypertrophic zones of growth plates were stained *via* IHC to detect type X collagen (Col-X) in 8-week-old mice. Scale bars, 50 μm. **(E)** Thicknesses of type X collagen-stained hypertrophic zone were quantified. **(F)** Thicknesses of growth plate and primary spongiosa of 4-week-old mice were measured by μ-CT analysis. **(G)** Lengths of epiphysis and metaphysis plus diaphysis of 8-week-old mice were measured by μ-CT analysis. Each group, *N* = 4–8. ^∗^*P* < 0.05, ^∗∗^*P* < 0.01, ^∗∗∗^*P* < 0.001. RZ: resting zone, PZ: proliferative zone, HZ: hypertrophic zone.

To analyze the effects of GPER-1 deficiency on tibial growth plate morphology, Safranin O/Fast Green staining for glycosaminoglycan was performed, which showed the structure of the growth plate in 8-week-old mice (Figure 4A). Histological analyses demonstrated that the cell numbers of growth plate were significantly decreased in the CKO mice compared with those in the control mice (Figure 4B). Morphometric analyses showed that the resting zones of the growth plate in the knockout mice had a similar size as seen in the control mice. In contrast, the thickness of the proliferative zones was decreased, and the hypertrophic zones were increased in the *GPER-1* knockout mice compared with those in the control mice (Figure 4C).

Histological analysis of type X collagen staining in the tibial growth plate cartilage showed a greater type X collagen distribution in the CKO mice compared with the control group (Figure 4D). We analyzed the hypertrophic region of the growth plate with the type X collagen expressed in the territorial matrix of hypertrophic chondrocytes. Morphometric analyses showed that GPER-1 deficiency increased the hypertrophic zone thickness (Figure 4E).

Hypertrophic chondrocytes are replaced by bone *via* apoptosis and remodel the metaphysis of the growing bone. The area containing a basophilic core of mineralized cartilage spicules and early ossification is termed the primary spongiosa. In 4-week-old mice, there was no significant difference in growth plate thickness between the control and knockout mice as determined *via* μ-CT analysis, but the thickness of the primary spongiosa was significantly reduced in CKO mice (Figure 4F). The epiphysis length showed no significant change between the control and CKO mice, but the lengths of the metaphysis plus diaphysis were significantly reduced in the 8-week-old CKO mice compared with that in the control (Figure 4G). These data indicated that the reduced bone length might be associated with a reduction in endochondral ossification because the primary spongiosa thickness and the lengths of metaphysis plus diaphysis were significantly reduced rather than that of the epiphysis.

### Effects of Chondrocyte-Specific G-Protein-Coupled Estrogen Receptor-1 Deficiency on the Chondrocyte Proliferation at Tibia Growth Plates

**FIGURE 5:**
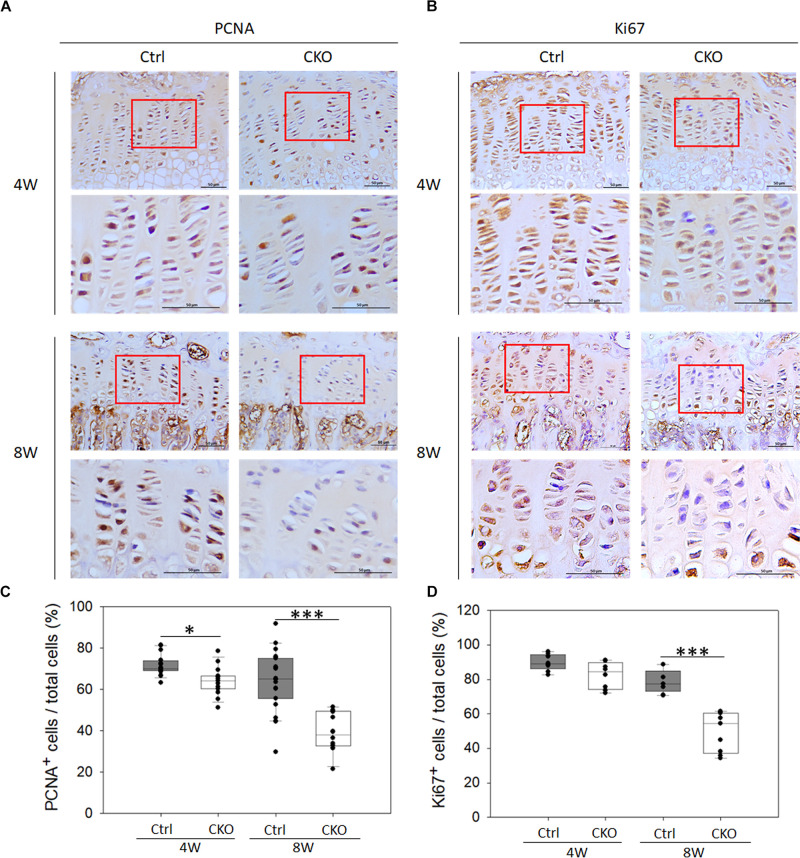
Effects of chondrocyte-specific GPER-1 knockout (CKO) on chondrocyte proliferation in tibia growth plates. **(A,B)** IHC staining of PCNA and Ki67 in tibial growth plates in 4- and 8-week-old female mice. Representative micrographs of growth plates at low and high magnification. Scale bars, 50 μm. **(C,D)** Quantification of ratio of proliferative cells to total cells was shown as ratio of PCNA- and Ki67-positive cells to hematoxylin-stained cells (total cells). Each group, *N* = 5. ^∗^*P* < 0.05, ^∗∗∗^*P* < 0.001.

To confirm whether the decreased growth plate thickness was due to suppressing chondrocyte proliferation in CKO mice, IHC analysis of PCNA and Ki67 was performed (Figure 5). The number of PCNA-positive cells per total cell in the growth plate was significantly reduced by GPER-1 deficiency in 4- and 8-week-old mice (Figure 5A). In 8-week-old mice, the number of Ki-67-positive cells also decreased in the CKO mice compared with that in the control group (Figure 5B). These data indicated that GPER-1 deficiency could reduce proliferative zone thickness and cell number which might be associated with the inhibition of chondrocyte proliferation.

### Effects of G-Protein-Coupled Estrogen Receptor-1 Agonist and Antagonist Treatment on Chondrocyte Proliferation in the Cultured Epiphyseal Cartilage

**FIGURE 6:**
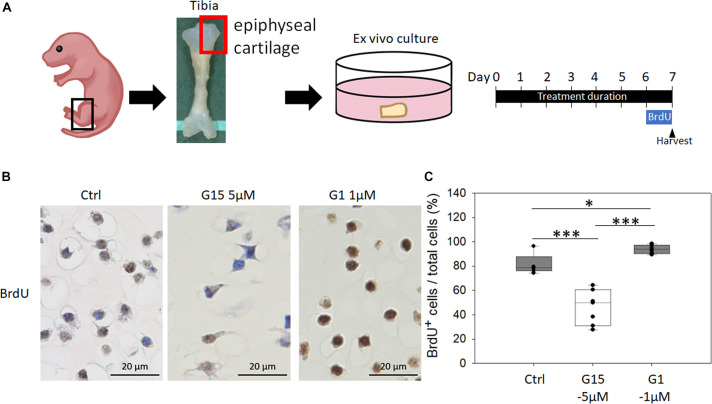
Effects of GPER-1 agonist and antagonist treatment on chondrocyte proliferation in cultured epiphyseal cartilage. **(A)** Graphical depiction of procedures described using *ex vivo* epiphyseal cartilage. **(B,C)** Antagonist (G15) and agonist (G1) of GPER-1 were used to testing effect of GPER-1 mediation on cell proliferation by measuring number of BrdU-positive chondrocytes. Scale bars, 20 μm. Each group, *N* = 5. ^∗^*P* < 0.05, ^∗∗∗^*P* < 0.001.

To analyze whether blocking GPER-1 expression reduces chondrocyte proliferation in cultured epiphyseal cartilage of tibias, we treated the epiphyseal articular cartilages with the specific antagonist (G15) and agonist (G1) of GPER-1 (Figure 6A). The results revealed fewer BrdU-positive cells in the G15 treatment group and more BrdU-positive cells in the G1 treatment group compared with the control group (Figures 6B,C). These findings showed that GPER-1 antagonists reduced chondrocyte proliferation in cultured articular cartilages. On the other hand, GPER-1 promoted chondrocyte proliferation.

## Discussion

This study is the first to investigate the role of GPER-1 in growth plate chondrocytes and its subsequent effect on bone growth at puberty using a tissue-specific *GPER-1* knockout mouse model. We originally demonstrated that GPER-1 deficiency results in reducing the chondrocyte proliferation, cell number, and thickness of proliferation zone in the growth plates of tibias in pubertal female mice. The reduction in the lengths of primary spongiosa, metaphysis, and diaphysis was also found in the tibias of CKO mice. Estrogen is known to be an important hormone in regulating long bone elongation during puberty. Circulatory levels of estrogen were thought to be a key factor in determining the growth and closure of the growth plate (Grumbach, 2004), however, the ERs that mediate this event remain unclear. In this study, our histological analysis found that the peak level of GPER-1 in growth plates was at early puberty, and then, it declined by age to extremely low levels at sexual maturation in the control mice. On the other hand, we also found that ERα expression has a marked increase at early puberty and is maintained until sexual maturation in female mice. This indicated that the existence and amount of GPER-1 might play a role in mediating estrogen signals to regulate chondrocyte proliferation and subsequent bone elongation during pubertal bone growth.

According to previous reports, the 2-, 4-, 8-, 12-, and 16-week-old mice represent the stages of early life, early-puberty, puberty, end-puberty, and post-puberty, respectively (van der Eerden et al., 2000; Li et al., 2012; Bell, 2018). In this study, we found that the percentage of GPER-1-positive cells in growth plates increased from the age of 2 to 4 weeks in mice but declined gradually to extremely low levels until the age of 16 weeks (from 60.61 to 9.47%). On the other hand, unlike the huge change in GPER-1, the percentage of ERα-positive cells in growth plates showed no significant change during puberty (from 4 to 16 weeks old). Other studies had also indicated that the expression of ERα protein in the growth plate did not significantly decline during sexual maturation in rats (van der Eerden et al., 2002; Li et al., 2012). In the human growth plates, ERα distribution and the percentage of Erα-positive cells showed no significant changes from childhood to adolescence (Egerbacher et al., 2002; Nilsson et al., 2003). Previous reports and the findings from this study indicate that GPER-1 might be involved in the modulation of bone growth at puberty rather than post-puberty. The most important event of pubertal bone growth should be the growth plate-involved bone elongation, in which chondrocytes play the most important role. Therefore, we used the CKO mice to study the role of GPER-1 in growth plate chondrocytes without affecting other cells in bones.

In this study, we generated novel CKO mice with floxed exon 3 of the *GPER-1* locus and the knockout of *GPER-1* in type II collagen-expressing tissue, resulting in chondrocyte-specific knockout (Figure 2A). We confirmed that GPER-1 was deficient in tissues expressing type II collagen, including the tibia growth plate, articular cartilage, and costal cartilage, in CKO mice (Figure 2E). Serum estrogen levels exhibited no difference between the CKO and control mice, which is similar to that found in female global *GPER-1* knockout mice in a previous study (Martensson et al., 2009). There have been several reports on the interaction between GPER-1 and nuclear ERs (Kang et al., 2010; Smith et al., 2016; Romano and Gorelick, 2018). A study indicated that GPER-1 might crosstalk with other ERs (Romano and Gorelick, 2018). Other studies on different cell lines found that a selective GPER-1 agonist inhibits nuclear ERs activity in human breast cancer cells (MCF-7) (Smith et al., 2016), whereas it was indicated to upregulate ERα expression in human breast cancer cells (SK-BR-3), human embryonic kidney cells (HEK293), and monkey kidney cells (COS7) (Kang et al., 2010). In this study, the ERα level in growth plate cartilages of CKO mice showed no difference between the control mice. This result indicates that the CKO system generated in this study specifically knocks out GPER-1 but is not interrupted by the estrogen ligand or the ERα crosstalk.

Longitudinal bone growth is through the process of endochondral bone formation. During this process, the growth plate chondrocytes undergo proliferation, hypertrophy, and eventually apoptosis. In this study, we found a decrease in the number of proliferative chondrocytes, length of proliferation zone of the growth plate, and length of the tibia in CKO mice. A previous study, using a global *GPER-1* knockout model with a deletion of a whole *GPER-1* open-reading frame, found that the lengths of femur and crown–rump were decreased in the female mice (Martensson et al., 2009). In contrast, a study on full *GPER-1* knockout male mice showed increased body length, bone mineral density, trabecular bone volume, and cortical bone thickness (Ford et al., 2011). These results indicated that a sexually dimorphic effect of GPER-1 might occur in global knockout mice. Additionally, in the global GPER-1 knockout model, systemic effects cannot be excluded, such as increased fat mass (Haas et al., 2009), increased blood pressure, and impaired glucose tolerance (Martensson et al., 2009). On the other hand, the tissue-specific GPER-1 knockout model can reduce the complexity of systemic interactions. In this study, using our CKO model, we demonstrated that CKO mice did not only have decreased bone length but also have decreased the number of Ki67- and PCNA-positive proliferative chondrocytes in the growth plate of 8-week-old pubertal female mice. These results emphasized that GPER-1 plays a crucial role in promoting the proliferation of growth plate chondrocytes and contributes to bone elongation during pubertal bone growth.

The length of the long bone is determined by increasing not only the height but also the timing of growth plates closure. In this study, we found that the number of GPER-1-positive chondrocytes was significantly lower in the post-puberty compared with the puberty stage, suggesting the bone elongation might terminate in adult mice. In contrast to the results found in the pubertal mice, we demonstrated that the bone lengths in femurs and tibia were shorter but not statistically different in the post-puberty (12-week-old) CKO mice compared with those in the control mice (Figures 3D,E). A previous study also indicated that estrogen treatment on the 12-week-old ovariectomized female *GPER*-1 knockout mice did not affect both the longitudinal skeletal growth and growth plate height (Windahl et al., 2009). Another report indicated that treatment of GPER-1 agonist on the 12-week-old ovariectomized female mice did not affect tibia and femur growth (Iravani et al., 2019). These two reports indicated that GPER-1 did not affect growth plate thickness in adult mice. Together with the results from these previous and current studies, the level change of GPER-1 expression may determine bone elongation rather than that of receptor ligands, such as estrogen and GPER-1 agonist.

Although estrogen and its receptors are involved in bone growth, the physiological regulation of bone growth and remodeling at any stage in life is dynamic and complicated. In previous GPER-1 studies, some of the conflicting results for bone growth might be because of differences in age, sex, and genetic backgrounds of the various animal models (Martensson et al., 2009; Ford et al., 2011). Furthermore, hormone regulation of longitudinal bone growth through endochondral ossification is also complicated. In addition to estrogen, longitudinal bone growth is also regulated by the growth hormone and thyroid hormone (Nilsson et al., 1994). Because both ERα and GPER-1 are expressed in growth plate chondrocytes, it is difficult to distinguish the effect *via* ERα, ERβ, or GPER-1 using the natural ligand estradiol. Therefore, we performed an *ex vivo* study to investigate the influence of GPER-1 on the cultured epiphyseal cartilage from the tibia upon treatment with a GPER-1-specific agonist (G1) and antagonist (G15). The results revealed that G1 significantly enhanced chondrocyte proliferation, whereas G15 showed an inhibitory effect. These findings are consistent with those of a recent study demonstrating the role of GPER-1 in increasing chondrocyte proliferation (Fan et al., 2018). Furthermore, an *ex vivo* study, which excluded the effects of complicated systemic factors, revealed that GPER-1 directly promotes chondrocyte proliferation, further confirming the *in vivo* findings.

The limitation of this study is the lack of investigation regarding the underlying molecular mechanisms at the cellular level. In our previous study, GPER-1 was found to mediate bone marrow-derived mesenchymal stem cell proliferation *via* the cyclic adenosine 3′,5′-monophosphate/protein kinase A/phosphorylation of cyclic adenosine 3′,5′-monophosphate-response element-binding protein pathway upon treatment with a GPER-1 agonist (Chuang et al., 2020). Another study indicated that the GPER-1 agonist activated the phosphoinositide 3-kinase/Akt signaling pathway in the ATDC5 cell line (Fan et al., 2018), however, it failed to induce Akt or ERK1/2 phosphorylation in human adult articular chondrocytes (Ribeiro et al., 2020). These studies showed that different GPER-1 mediated signal pathways might occur in different types of cells. Accordingly, the intracellular regulatory mechanism of GPER-1 in grow plate chondrocytes is also worth investigating. In conclusion, chondrocyte-specific *GPER-1* knockout in female mice and subsequent treatment with a specific GPER-1 agonist or antagonist on the implant culture of epiphytical cartilage showed that GPER-1 plays an important role in facilitating the elongation of long bone by enhancing the growth plate chondrocyte proliferation in female pubertal mice.

## Data Availability Statement

The original contributions presented in the study are included in the article/Supplementary Material, further inquiries can be directed to the corresponding author/s.

## Ethics Statement

The animal study was reviewed and approved by Kaohsiung Medical University Animal Care and Use Committee (104166 and 107157).

## Author Contributions

All authors listed have made a substantial, direct and intellectual contribution to the work, and approved it for publication.

## Conflict of Interest

The authors declare that the research was conducted in the absence of any commercial or financial relationships that could be construed as a potential conflict of interest.

## Publisher’s Note

All claims expressed in this article are solely those of the authors and do not necessarily represent those of their affiliated organizations, or those of the publisher, the editors and the reviewers. Any product that may be evaluated in this article, or claim that may be made by its manufacturer, is not guaranteed or endorsed by the publisher.
