# Supplementary figures and images for: G-Protein-Coupled Estrogen Receptor-1 Positively Regulates the Growth Plate Chondrocyte Proliferation in Female Pubertal Mice
Source: Front Cell Dev Biol. 2021 Aug 20;9:710664. doi: 10.3389/fcell.2021.710664 (PMC8417792; doi:10.3389/fcell.2021.710664)

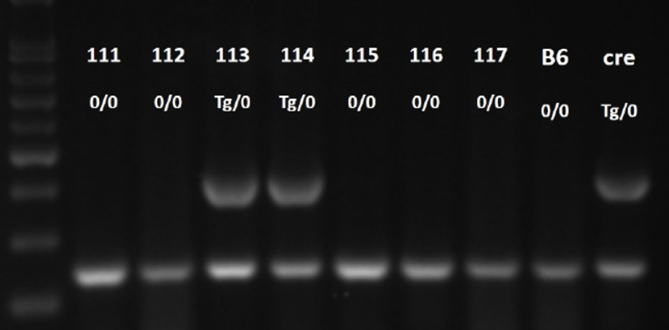

Supplement: Supplementary file 1 [file Image_1.TIF]

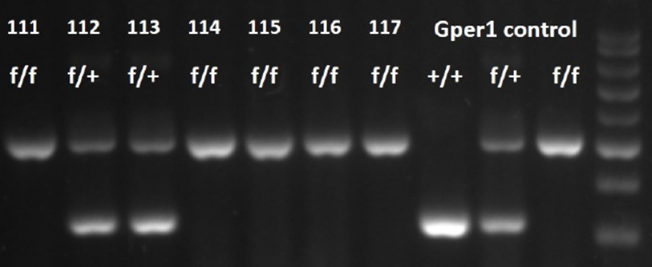

Supplement: Supplementary file 2 [file Image_2.TIF]
